# Association of serum magnesium level with small fiber neuropathy in patients with type 2 diabetes

**DOI:** 10.3389/fmed.2025.1509820

**Published:** 2025-03-04

**Authors:** Xiaoting Liu, Jianzhang Hu

**Affiliations:** ^1^Department of Ophthalmology, Fujian Provincial Governmental Hospital, Fuzhou, Fujian, China; ^2^Department of Ophthalmology, Fujian Medical University Union Hospital, Fuzhou, Fujian, China

**Keywords:** serum magnesium, corneal nerve fibers, small fiber neuropathy, type 2 diabetes mellitus, corneal confocal microscopy

## Abstract

**Purpose:**

We aimed to investigate the association between serum magnesium (Mg) levels and small fiber neuropathy among patients with type 2 diabetes mellitus (T2DM).

**Methods:**

This study retrospectively collected data from patients with T2DM. Patients were divided based on the quartiles of the serum concentrations of Mg. Corneal confocal microscopy (CCM) was employed to determine the morphological parameters of corneal nerve fibers, including corneal nerve fiber length (CNFL), fiber density (CNFD), and branch density (CNBD). Pearson correlation analysis and multiple linear regression analyses were conducted to investigate the association between the serum levels of Mg and the morphological parameters of corneal nerve fibers.

**Results:**

In total, 136 patients with T2DM were enrolled in this study. All morphological parameters of corneal nerve fibers increased with the increasing quartiles of serum Mg levels. Using Pearson correlation analysis, we found a significant and positive association between the serum levels of Mg and CNFL (*r* = 0.550, *p* < 0.001), CNFD (*r* = 0.432, *p* < 0.001), and CNBD (*r* = 0.425, *p* < 0.001). After adjusting for covariates, the serum levels of Mg remained positively correlated with CNFL (*β* = 0.495, *p* < 0.001), CNFD (*β* = 0.361, *p* < 0.001), and CNBD (*β* = 0.374, *p* < 0.001) in the fully adjusted model.

**Conclusion:**

The serum levels of Mg were positively and independently correlated with the morphological parameters of the corneal nerve among patients with T2DM. Serum Mg levels can serve as a potential biomarker for screening corneal small fiber neuropathy in patients with T2DM.

## Introduction

Corneal nerves primarily originate from the ophthalmic branch of the trigeminal nerve. They play a crucial role in maintaining the structural integrity and healthy function of the ocular surface by releasing neurotrophic factors (1). Long-term hyperglycemia in patients with diabetes can lead to corneal neuropathy. Corneal neuropathy is a type of small fiber neuropathy affecting the corneal nerves in patients with diabetes. It is characterized by corneal hypoesthesia, photophobia, ocular irritation or pain (2). Without timely diagnosis and treatment, it may lead to irreversible vision loss.

Corneal confocal microscopy (CCM) is a rapid and non-invasive technique that can help evaluate the morphology of small nerve fibers in a real-time manner (3, 4). It enables longitudinal studies of small nerve fibers in both animal models (5) and humans (6). It is an invaluable tool for detecting and monitoring neurodegenerative changes in patients with diabetes (7). CCM showed a significant decrease in the corneal nerves of patients with type 1 or type 2 diabetes (8, 9). Decreased corneal nerve fiber length (CNFL) and corneal nerve fiber density (CNFD) are the characteristic features of small fiber neuropathy (10).

Although CCM can effectively assess small fiber neuropathy, its use in clinical practice is limited by several factors, such as high costs, low availability, and the need for skilled professionals. In addition, most patients with diabetes receive care in primary health care facilities, where there are usually no professional ophthalmologists or CCMs. Therefore, a brief, low-cost, and easily accessible screening marker is needed to diagnose diabetic corneal neuropathy.

Magnesium (Mg) ion is the fourth most abundant cation in the body (11), which plays a critical role in stabilizing blood glucose levels (12). There is a consensus that hypomagnesemia is closely associated with the development of diabetes mellitus (DM). Previous studies have shown that low serum level of Mg is an independent risk factor for heart failure, atrial fibrillation, diabetic nephropathy, and retinopathy in patients with type 2 diabetes mellitus (T2DM) (13). Recent studies have found that low serum levels of Mg are associated with peripheral neuropathy in patients with T2DM (14, 15). Small fiber neuropathy is generally considered an early manifestation of peripheral neuropathy (16); therefore, we hypothesized that there may be a similar association between the serum levels of Mg and small fiber neuropathy. This study explored the association between serum Mg levels and small fiber neuropathy among patients with T2DM.

## Methods

### Patients

Between December 2020 and December 2021, this retrospective, cross-sectional study was conducted at the Department of Ophthalmology, Fujian Medical University Union, Fuzhou, China. This study included patients with T2DM who underwent CCM. Patients were divided into four groups based on the quartiles of the serum concentrations of Mg. Patients were excluded if they had a history of malignancy, autoimmune diseases, wearing a contact lens, ocular trauma, any ocular surgery, or any other ocular or systemic diseases (except for T2DM) affecting the cornea. This study followed the principles of the Declaration of Helsinki and was approved by the Ethics Committee of the Fujian Medical Union.

### Clinical and ophthalmologic assessments

Participants underwent a comprehensive medical assessment. Their body mass index (BMI), blood pressure (BP), fasting plasma glucose (FPG), glycated hemoglobin (HbA1c), serum Mg, total cholesterol (TC), triglyceride (TG), LDL-cholesterol (LDL-c), HDL-cholesterol (HDL-c), fasting C-peptide, and serum creatinine (SCr) levels were measured. Kidney function was assessed by estimating the glomerular filtration rate (eGFR). A thorough ophthalmic examination was done, including slit-lamp biomicroscopy, measurement of intraocular pressure, evaluation of dilated fundus, and CCM examination.

### Corneal confocal microscopy

CCM examination was conducted using laser scanning confocal microscopy (Heidelberg Retinal Tomograph III Rostock Cornea Module; Heidelberg Engineering GmbH, Heidelberg, Germany) as described previously (17). All CCM examinations were conducted by a trained examiner. For each eye of each patient, three representative images of the central corneal sub-basal nerve plexus were selected and analyzed based on their quality, depth, optimal contrast, and focus position. The images were analyzed using validated and established automatic nerve analysis software (ACCMetrics; University of Manchester, Manchester, United Kingdom) (18). We quantified three morphological parameters of the corneal nerve: (1) CNFL, which represents the total length of all nerve fibers and branches (mm per 1 mm^2^); (2) CNFD, which indicates the total number of major nerves per 1 mm^2^; and (3) corneal nerve branch density (CNBD), which represents the number of branches emanating from major nerve trunks per 1 mm^2^.

### Statistical analysis

The statistical analysis was conducted using SPSS (version 26; SPSS, Chicago, IL) and R software (version 4.2.3, R Foundation, Vienna, AUS). Continuous data are presented as mean ± standard deviation or median (interquartile range), while categorical data are presented as number (%). One-way analysis of variance (ANOVA) followed by Games-Howell’s *post hoc* test was used to compare normally distributed data. Non-normally distributed data were compared using the Kruskal-Wallis test. The Chi-square test was employed to compare categorical variables. Pearson correlation and multiple linear regression analysis were conducted to analyze the association between serum Mg levels and the morphological parameters of the corneal nerve. Covariates were selected based on previous studies (19–21) and clinical experience. Specifically, the following covariates were used in the regression models: age, gender, diabetes duration, BMI, SBP, HbA1c, FBG, triglyceride, TC, eGFR, and fasting C-peptide. Robust regression analysis was conducted to evaluate the robustness of the findings. Additionally, partial correlation analysis was conducted to determine the associations between metabolic variables and the morphological parameters of the corneal nerve, while controlling for age and sex. *p* < 0.05 was considered statistically significant.

## Results

### Demographic and clinical data

**Figure 1:**
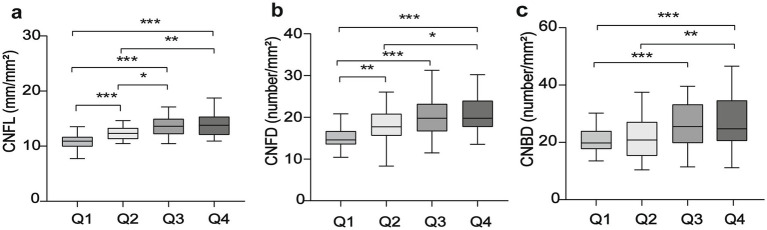
Differences in CNFL **(a)**, CNFD **(b)**, and CNBD **(c)** between groups (Q1–4) based on the quartile of serum Mg levels. Statistical differences between groups are indicated by *p* values (^*^p < 0.05, ^**^ *p* < 0.01, and ^***^ *p* < 0.001).

**Figure 2:**
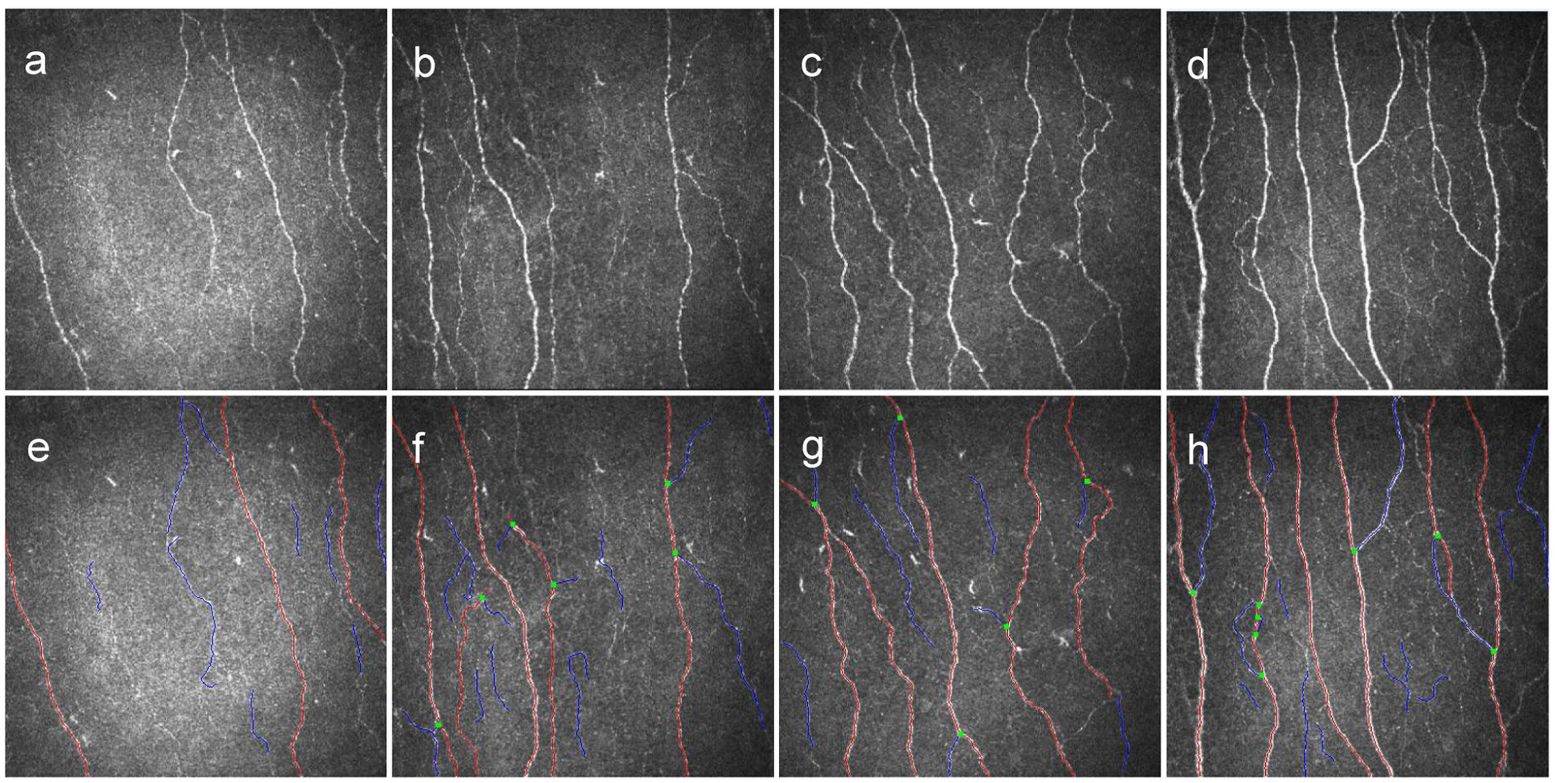
Original and annotated representative images of the sub-basal nerve plexus in the Q1 group **(a,e)**, Q2 group **(b,f)**, Q3 group **(c,g)**, and Q4 group **(d,h)** from age-matched patients. Red, fiber; blue, branch; green, branch point.

In total, 136 patients (272 eyes) diagnosed with T2DM were included in this study. Their demographic and clinical data are presented in Table 1. Participants were divided into four groups (Q1–Q4) based on the quartiles of the serum concentrations of Mg: Q1: Mg < 0.81 mmol/L; Q2: 0.81 mmol/L ≤ Mg < 0.86 mmol/L; Q3: 0.86 mmol/L ≤ Mg < 0.91 mmol/L; and Q4: Mg ≥ 0.91 mmol/L. We found a significant difference in FBG (*p* < 0.020), CNFD (*p* < 0.001), and CNBD (*p* < 0.001) among the four groups (Table 1). However, no significant differences were identified concerning other variables. Furthermore, among the four groups, all morphological parameters, including CNFL, CNFD, and CNBD, increased with the increase in the serum levels of Mg (Figures 1, 2).

**Table 1:** Demographic and clinical characteristics of participants by serum magnesium levels quartile.

	Q1 (*n* = 34)	Q2 (*n* = 34)	Q3 (*n* = 34)	Q4 (*n* = 34)	*p*
Male (*n* (%))	64.7	64.7	61.8	61.8	0.988
Age (years)	61 ± 11.75	57.53 ± 11.44	56.76 ± 12.50	60.74 ± 10.62	0.315
BMI (kg/m^2^)	23.1 ± 3.85	24.89 ± 4.06	24.73 ± 3.38	24.60 ± 3.41	0.196
Duration of diabetes (years)	12 (5–20)	13 (5–20)	7.5 (2–18)	8.5 (4–14)	0.118
SBP (mmHg)	132.68 ± 16.69	132.94 ± 15.13	129.94 ± 18.53	132.68 ± 16.69	0.844
DBP (mmHg)	80.29 ± 10.67	82.68 ± 12.44	80.88 ± 9.92	80.29 ± 10.67	0.675
FBG (mmol/L)	8.40 ± 3.62	8.43 ± 2.88	7.92 ± 2.78	6.49 ± 1.67	0.020
HbA1c (%)	7.40 (8.60–10.90)	7.50 (8.30–9.70)	7.80 (8.90–10.20)	7.20 (8.05–9.10)	0.347
Fasting C-peptide (ng/mL)	0.59 (0.41–0.83)	0.68 (0.42–0.92)	0.68 (0.43–0.80)	0.81 (0.53–1.08)	0.239
TG (mmol/L)	1.55 (1.17–2.35)	1.55 (1.12–2.56)	1.71 (1.10–2.17)	1.96 (1.16–2.62)	0.889
TC (mmol/L)	1.17 (1.55–2.35)	1.12 (1.55–2.56)	1.10 (1.71–2.17)	1.16 (1.96–2.62)	0.696
HDL (mmol/L)	1.08 (0.94–1.16)	1.05 (0.90–1.46)	1.00 (0.80–1.31)	1.01 (0.89–1.19)	0.558
LDL (mmol/L)	2.89 ± 1.26	3.09 ± 0.97	3.03 ± 0.96	2.93 ± 1.07	0.864
Cr (μmol/L)	63.50 (54.00–76.00)	67.00 (60.00–79.00)	66.50 (56.00–82.00)	73.00 (66.00–84.00)	0.126
eGFR (mL/min/1.73m^2^)	92.80 (81.40–101.00)	89.55 (80.80–101.20)	97.10 (86.90–106.90)	96.35 (81.40–104.70)	0.393
CNFL (mm/mm^2^)	10.83 ± 1.39	12.35 ± 1.15^a^	13.50 ± 1.72^a^^,^^b^	13.90 ± 2.06^a^^,^^b^	<0.001
CNFD (number/mm^2^)	15.13 ± 2.78	17.89 ± 3.80^a^	19.85 ± 4.41^a^	20.63 ± 4.20^a^^,^^b^	<0.001
CNBD (number/mm^2^)	18.44 ± 5.47	21.41 ± 7.05	26.02 ± 8.55^a^	29.04 ± 9.69^a^^,^^b^	<0.001

Data are presented as mean ± SD or median (interquartile range). BMI, body mass index; BP, blood pressure; FBG, fasting blood glucose; HDL, high-density lipoprotein; LDL, low-density lipoprotein; TC, total cholesterol; TG, triglyceride; eGFR, estimated glomerular filtration rate. Statistical differences between groups are represented by the *p* values.

a Significant difference compared with quartile 1.

b Significant difference compared with quartile 2.

### Correlation analysis

**Figure 3:**
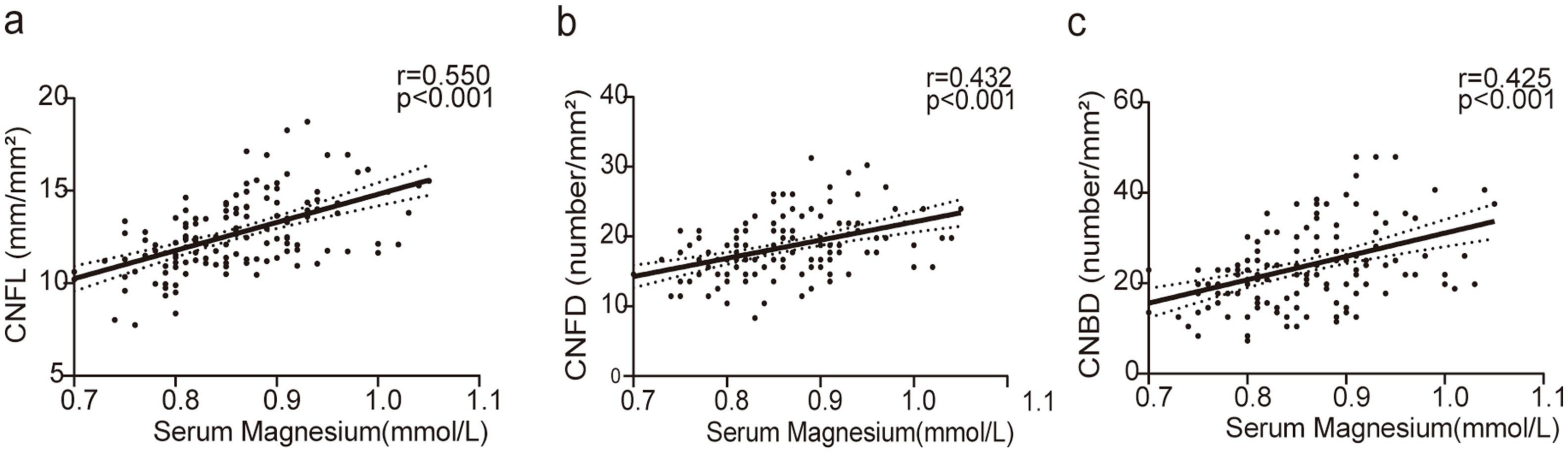
The associations between the serum levels of magnesium and morphological parameters of corneal nerve fibers. **(a)** Pearson correlation analysis revealed a strong association between serum magnesium levels and CNFL (*r* = 0.550, *p* < 0.001). **(b,c)** There was a moderate association between serum magnesium levels and CNFD (*r* = 0.432, *p* < 0.001) and CNBD (*r* = 0.425, *p* < 0.001).

Pearson correlation analysis was conducted to assess the association between the serum levels of Mg and the morphological parameters of the corneal nerve. Our results revealed a strong positive association between the serum levels of Mg and CNFL (*r* = 0.550, *p* < 0.001) (Figure 3a). A moderate association was observed between the serum levels of Mg and CNFD (*r* = 0.432, *p* < 0.001; Figure 3b) and CNBD (*r* = 0.425, *p* < 0.001; Figure 3c). Considering age and gender as control variables, we conducted a partial correlation analysis to investigate the association between the serum levels of Mg, other metabolic indicators, and the morphological parameters of the corneal nerve. The serum levels of Mg remained positively correlated with CNFL, CNFD, and CNBD (Table 2). Furthermore, our findings revealed that FBG was negatively correlated with CNFL (*r* = −0.225, *p* = 0.009), CNFD (*r* = −0.179, *p* = 0.039), and CNBD (*r* = −0.215, *p* = 0.012). In contrast, fasting C-peptide was positively correlated with CNFL (*r* = 0.242, *p* = 0.005), CNFD (*r* = 0.247, *p* = 0.004), and CNBD (*r* = 0.215, *p* = 0.013). Furthermore, HbA1c was inversely correlated with CNFL (*r* = −0.181, *p* = 0.036). Besides, the duration of diabetes was negatively correlated with CNFD (*r* = −0.180, *p* = 0.037). Moreover, BMI was positively correlated with both CNFD and CNBD (*r* = 0.176, *p* = 0.042; *r* = 0.201, *p* = 0.020), and HDL-c was inversely correlated with CNFL and CNBD (*r* = −0.173, *p* = 0.046; *r* = −0.171, *p* = 0.049).

**Table 2:** Partial correlation analysis of corneal nerve fibers morphological parameters with clinical and serologic data (adjusted for age and gender).

Characteristics	CNFL		CNFD		CNBD	
	*r*	*p*	*r*	*p*	*r*	*p*
BMI	0.165	0.056	0.176	0.042	0.201	0.020
Duration of diabetes	−0.133	0.126	−0.180	0.037	−0.008	0.930
SBP	0.029	0.736	0.037	0.673	0.021	0.814
DBP	0.024	0.780	0.015	0.864	0.045	0.605
FBG	−0.225	0.009	−0.179	0.039	−0.215	0.012
HbA1c	−0.181	0.036	−0.125	0.149	−0.140	0.107
Fasting C-peptide	0.242	0.005	0.247	0.004	0.215	0.013
TG	−0.008	0.930	−0.022	0.805	−0.005	0.954
TC	−0.109	0.211	−0.100	0.252	−0.025	0.778
HDL	−0.173	0.046	−0.168	0.052	−0.171	0.049
LDL	−0.109	0.209	0.046	0.594	−0.031	0.726
Cr	0.170	0.050	0.146	0.092	0.156	0.072
eGFR	−0.060	0.491	−0.038	0.660	−0.030	0.734
Serum Mg	0.553	<0.001	0.427	<0.001	0.424	<0.001

Finally, multiple linear regression analysis was employed to identify independent relationships between the serum levels of Mg and the morphological parameters of the corneal nerve. The outcomes of this analysis are expressed as standardized regression coefficients (*β*) (Table 3). The results demonstrated a significant association between the serum levels of Mg and CNFL (*β* = 0.550, *p* < 0.001), CNFD (*β* = 0.432, *p* < 0.001), and CNBD (*β* = 0.425, *p* < 0.001) before adjustments (model 1). After controlling for age, sex, and duration of diabetes (model 2), we found a positive association between the serum levels of Mg and CNFL (*β* = 0.546, *p* < 0.001), CNFD (*β* = 0.408, *p* < 0.001), and CNBD (*β* = 0.434, *p* < 0.001). The association between the serum levels of Mg and CNFL (*β* = 0.495, *p* < 0.001), CNFD (*β* = 0.361, *p* < 0.001), and CNBD (*β* = 0.374, *p* < 0.001) remained significant even after adjusting for BMI, SBP, HbA1c, FBG, TG, TC, eGFR, and fasting C-peptide (model 3). Additionally, robust regression analysis, which accounted for the effect of outliers, also confirmed a significant association between the serum levels of Mg and each morphological parameter of the corneal nerve.

**Table 3:** Multiple linear regression (MLR) analysis and robust regression of serum magnesium levels with corneal nerve fibers morphological parameters (as dependent variable).

Models	Characteristics	CNFL				CNFD				CNBD			
		MLR		Robust		MLR		Robust		MLR		Robust	
		*β*	*p* value	*β*	*p* value	*β*	*p* value	*β*	*p* value	*β*	*p* value	*β*	*p* value
Model 1	Serum Mg	0.550	<0.001	0.526	<0.001	0.432	<0.001	0.408	<0.001	0.425	<0.001	0.398	<0.001
Model 2	Serum Mg	0.546	<0.001	0.525	<0.001	0.408	<0.001	0.386	<0.001	0.434	<0.001	0.408	<0.001
Model 3	Serum Mg	0.495	<0.001	0.476	<0.001	0.361	<0.001	0.348	<0.001	0.374	<0.001	0.349	<0.001

Model 1: a regression model including just serum magnesium.

Model 2: adjusted for serum magnesium, age, gender, diabetes duration.

Model 3: adjusted for serum magnesium, age, gender, diabetes duration, BMI, SBP, HbA1c, FBG, TG, TC, eGFR, and fasting C-peptide.

*β is the standardized coefficient.*

## Discussion

Patients with diabetes often present with corneal nerve damage, which affects the morphological parameters of the cornea, such as nerve fiber length, nerve density, and branching density (22). Additionally, corneal nerve damage may delay corneal wound healing and increase the risk of corneal infections (23). In this retrospective cross-sectional study, we found that the serum levels of Mg in patients with T2DM were positively associated with the morphological parameters of the corneal nerve (CNFL, CNFD, and CNBD) detected by CCM, a non-invasive imaging technique. After adjusting for potential confounding factors, we found that elevated Mg levels remained an independent protective factor for CNFL, CNFD, and CNBD. Although CCM allows for the visualization of small corneal nerve fibers, its application needs expertise and is limited in some institutions due to high costs. These findings strongly suggest that elevated serum levels of Mg can serve as an independent protective factor for corneal nerves. Given the ease and cost-effectiveness of measuring the serum levels of Mg through routine laboratory tests, this biomarker can be used as a practical tool for screening and monitoring small fiber neuropathy in patients with T2DM without imposing additional burdens.

Numerous epidemiological studies have shown that Mg is closely associated with diabetes and its complications (13). Mg may contribute to the development and progression of diabetes by affecting insulin resistance, insulin secretion, inflammatory responses, and oxidative stress (24, 25). Furthermore, Mg is essential for the survival and function of neurons (26). Recent studies have demonstrated a strong association between the serum levels of Mg and peripheral neuropathy associated with diabetes. For instance, the serum levels of Mg were significantly lower in patients with T2DM and abnormal nerve conduction compared to those with normal nerve conduction (15). Strom A et al. reported that Mg concentrations were reduced in patients with recently diagnosed type 2 diabetes and diabetic sensorimotor polyneuropathy (27). Consistently, Mg supplementation was shown to improve neurobehavioral and electrophysiological functions (28). In the present study, a significant positive association was observed between the serum levels of Mg and the morphological parameters of the corneal nerve. This finding offers potential guidance for the early detection, monitoring, and treatment of diabetic corneal neuropathy. Additionally, lower serum levels of Mg and corneal nerve damage have also been observed in various metabolic-related disorders, such as prediabetes, glycemic variability, and metabolic syndrome (29–33). Given the significant role of these conditions in the pathogenesis and progression of diabetes, monitoring the serum levels of Mg and corneal nerve damage in such patients is of great clinical importance.

Although the pathological mechanisms underlying diabetic corneal neuropathy are not yet fully known, accumulating evidence shows that the inflammatory response, oxidative stress, and intracellular inositol concentrations play critical roles in the pathogenesis of diabetic corneal neuropathy (34). The serum levels of Mg have been shown to possess anti-inflammatory and antioxidant properties (35). Decreased serum levels of Mg exacerbate oxidative stress and the inflammatory response (24, 25), primarily by attenuating oxidative stress kinases and upregulating pro-inflammatory factors, such as tumor necrosis factor-alpha (TNF-ɑ), interleukin-6 (IL-6), and interleukin-8 (IL-8) (36, 37). In addition, Zghoul et al. reported that Mg supplementation can downregulate pro-inflammatory factors (38). Moreover, Mg can increase the intracellular concentrations of inositol by increasing the intracellular transport of inositol, which helps prevent further neurological damage (39). It has been suggested that decreased levels of myoinositol can lower the activity of membrane sodium-potassium adenosine triphosphatase (Na+/K+ ATPase), finally impairing the function and structure of nerve fibers (40). Based on these findings, it can concluded that the neuroprotective effects of serum Mg are associated with its ability to increase the intracellular concentrations of inositol and attenuate inflammation and oxidative stress. However, further studies are necessary to fully elucidate the mechanisms underlying the effect of serum Mg in small fiber neuropathy.

We also observed a positive association between C-peptide levels and corneal nerve parameters in patients with T2DM, which is consistent with the findings of Zuo et al. (19). Previous studies demonstrated that the C-peptide level can be regarded as a reliable indicator of hyperinsulinemia and can help predict the risk of developing diabetes (41, 42). Qiao et al. (43) reported the protective effects of C-peptide on diabetic peripheral neuropathy. Tam et al. (44) indicated that the neuroprotective effects of C-peptide are linked to the mitogen-activated protein kinase pathways. Nevertheless, the role of C-peptide in corneal nerve function in patients with T2DM needs further studies. Meanwhile, HbA1c was inversely correlated with CNFL, and the duration of diabetes was negatively correlated with CNFD, consistent with the results of Pellegrini et al. (20) and Dehghani et al. (21). In addition, our data showed that BMI was positively correlated with CNFD and CNBD, while HDL-c was negatively correlated with CNFL and CNBD. Although high BMI and low HDL-c levels are considered risk factors for diabetes (45, 46). These inconsistencies may originate from differences in the study populations. Further studies are needed to investigate the reasons behind these discrepancies.

To the best of our knowledge, this study was the first to assess the association between the serum levels of Mg and small fiber neuropathy in individuals with T2DM. Our findings indicated a positive association between the serum levels of Mg and the morphological parameters of the corneal nerve. Therefore, the serum levels of Mg may serve as a potential indicator of small fiber neuropathy in individuals with T2DM. Our study provided a novel insight into the pathogenesis of small fiber neuropathy associated with diabetes. Despite strengths, it is crucial to acknowledge certain limitations to our study that warrant attention. Firstly, its retrospective nature could lead to selection bias. Secondly, due to the inherent nature of cross-sectional studies, we could not establish a causal relationship between the serum levels of Mg and corneal neuropathy. Thirdly, although we adjusted for several potential confounders, the possibility of residual confounding factors could not be completely ruled out. Finally, this study had a relatively small sample size. Therefore, large-scale population-based prospective and longitudinal studies are imperative to elucidate the association between the serum levels of Mg and corneal neuropathy. We intend to address these concerns in our future research endeavors.

## Conclusion

In summary, our study revealed that the serum levels of Mg were positively and independently associated with the morphological parameters of the corneal nerve in patients with T2DM. Therefore, the serum levels of Mg may serve as a potential biomarker for screening small fiber neuropathy among patients with T2DM. Future studies are needed to prospectively confirm our clinical findings and investigate the role of serum Mg in the pathogenesis of small fiber neuropathy in patients with T2DM.

## Data Availability

The raw data supporting the conclusions of this article will be made available by the authors, without undue reservation.
